# Association between Polygenic Risk Score and One-Year Outcomes Following As-Needed Aflibercept Therapy for Exudative Age-Related Macular Degeneration

**DOI:** 10.3390/ph13090257

**Published:** 2020-09-20

**Authors:** Taiyo Shijo, Yoichi Sakurada, Seigo Yoneyama, Wataru Kikushima, Atsushi Sugiyama, Mio Matsubara, Yoshiko Fukuda, Fumihiko Mabuchi, Kenji Kashiwagi

**Affiliations:** Department of Ophthalmology, University of Yamanashi, Shimokato 1110, Chuo, Yamanashi 409-3898, Japan; tshijoh@yamanashi.ac.jp (T.S.); syoneyama@yamanashi.ac.jp (S.Y.); wkikushima@yamanashi.ac.jp (W.K.); asugiyama@yamanashi.ac.jp (A.S.); miom@yamanashi.ac.jp (M.M.); ysugiyama@yamanashi.ac.jp (Y.F.); mabuchif-oph@umin.ac.jp (F.M.); kenjik@yamanashi.ac.jp (K.K.)

**Keywords:** age-related macular degeneration, polygenic risk score, aflibercept therapy, treatment outcomes, *ARMS2*, *CFH*

## Abstract

We investigated whether polygenic risk score (PRS) was associated with one-year outcome of as-needed aflibercept therapy for exudative age-related macular degeneration (AMD), including AMD (*n* = 129) and polypoidal choroidal vasculopathy (*n* = 132). A total of 261 patients were treated with as-needed intravitreal aflibercept injection (IAI) after three monthly IAIs and the completion of a one-year follow-up. One hundred and seventy-two healthy volunteers served as controls. Genotyping of *ARMS2* A69S (rs10490924), *CFH* I62V (rs800292), *SKIV2L-C2-CFB* (rs429608), *C3* (rs2241394), *ADAMTS-9* (rs6795735) and *CETP* (rs3764261) was performed for all participants. A total of 63 PRSs were quantified. There was a positive association between the PRS involving *ARMS2, CFH, C3*, *and ADAMTS-9* and best-corrected visual acuity at twelve months (*p* = 0.046, multiple regression analysis). When comparing PRSs of patients requiring retreatment and of patients without retreatment, 35 PRSs were significantly greater in patients requiring retreatment than in patients without requiring retreatment, with the PRS involving *ARMS2* and *CFH* being most significantly associated (*p* = 1.6 × 10^−4^). The number of additional injections was significantly associated with 40 PRSs and the PRS involving *ARMS2* and *CFH* showed a most significant *p*-value (*p* = 2.42 × 10^−6^). Constructing a PRS using a combination with high-risk variants might be informative for predicting the response to IAI for exudative AMD.

## 1. Introduction

Age-related macular degeneration (AMD), the leading cause of moderate and severe irreversible vision loss in people over 50 years of age worldwide, is a chronic inflammatory disease with a multifactorial etiology including the combined effects of multiple genes and environmental factors [1,2]. Of the implicated genetic factors, variants of *ARMS2* and *CFH* are major contributors to AMD pathogenesis. To date, the association of almost 20 genes with neovascular AMD has been demonstrated in Asian populations [3].

The advent of vascular endothelial growth factor (VEGF) inhibitors (bevacizumab, ranibizumab, aflibercept) revolutionized the treatment of exudative AMD. Worldwide, intravitreal administration of VEGF inhibitors is currently the first-line treatment option for exudative AMD. Aflibercept is a recombinant fusion glycoprotein binding to all isoforms of VEGF-A, VEGF-B, and placental growth factor with approximately 100 times higher binding affinity compared with bevacizumab and ranibizumab [4]. The VIEW1/2 study demonstrated that bimonthly intravitreal aflibercept injection (IAI) following an initial three-month IAI course is non-inferior to the monthly administration of ranibizumab regarding the improvement of best-corrected visual acuity as an endpoint [5]. *Pro re nata* (PRN) is an alternative regimen to monthly dosing for treating exudative AMD [6].

Several prospective studies demonstrated that visual improvement at twelve months was comparable following either monthly dosing or PRN dosing regimens [7,8]; however, some investigators have reported that the PRN regimen is less appropriate than a “treat and extend” regimen for best corrected visual acuity maintenance and improvement [9,10].

Recently, Heesterbeek et al. [11] demonstrated that a polygenic risk score (PRS) can contribute to predicting AMD progression (*n* = 177). However, to date, no reports describe research investigating the relationship between PRS and treatment outcomes in the context of VEGF-inhibitor-based treatment of exudative AMD.

Thus, the present study genotyped six major variants of *ARMS2, CFH, C2-CFB-SKIV2L, C3, ADAMTS-9, CETP* in order to calculate PRSs for each patient and investigate their association with one-year outcomes following as-needed aflibercept therapy for exudative AMD.

## 2. Results

A total of 261 patients (mean age: 74.8 ± 8.3 years, male: 72%) comprising 129 patients with neovascular AMD and 132 patients with polypoidal choroidal vasculopathy (PCV) were enrolled between January 2013 and July 2019. Table 1 shows the baseline demographic and genetic data of the participants. The mean age of the first subgroup was 77.1 ± 8.1 years and of the second was 72.5 ± 7.8 years. Thus, the second subgroup was significantly younger (*p* = 3.84 × 10^−6^). Males made up 66.7% of the first group and 77.3% of the second. Thus, no significant differences in gender distribution existed between the subgroups (*p* = 0.056). Risk allele frequency of *ARMS2*, *C3*, and *C2-CFB-SKIV2*L was significantly higher in AMD compared to PCV (*p* = 0.019, 0.02 and 0.03, respectively). Of the 63 possible combinations, 23 PRSs were significantly higher in AMD than in PCV. Table 2 shows the odds ratios of six genetic variants genotyped in the present study.

Mean logarithm minimum angle resolution (logMAR) BCVA significantly improved from 0.42 ± 0.37 at baseline to 0.27 ± 0.35 at twelve months (*p* = 1.5 × 10^−14^). Table 3 shows baseline factors associated with logMAR BCVA at twelve months. Of the 63 possible combinations, only the PRS involving *ARMS2*, *CFH*, *C3*, and *ADAMTS9* was significantly associated with BCVA at twelve months (*p* = 0.046).

**Figure 1:**
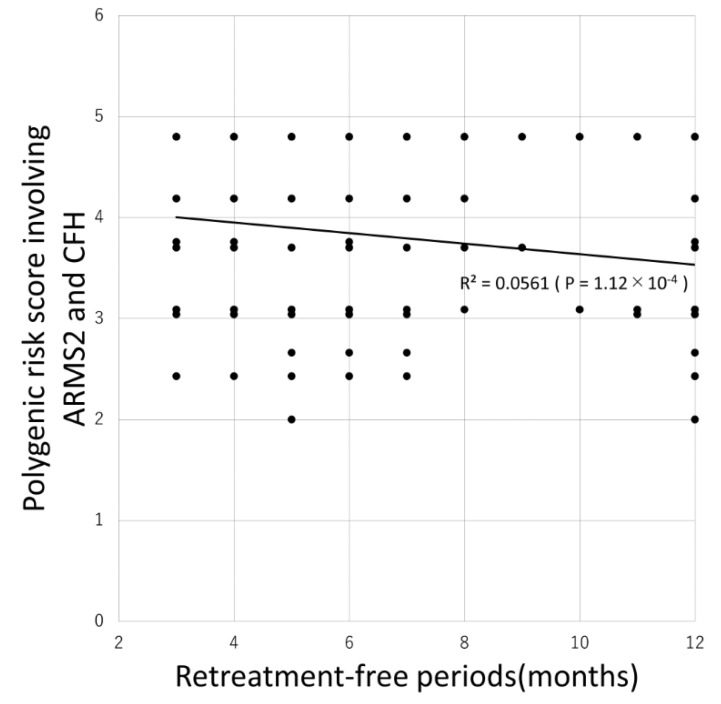
Association between retreatment-free period and polygenic risk score involving *ARMS2* and *CFH.*

Table 4 shows the comparison between patients requiring retreatment and without requiring retreatment. Of the 63 possible combinations, 35 PRSs were significantly associated with the requirement for retreatment with the PRS involving *ARMS2* and *CFH* being most significantly associated (*p* = 1.6 × 10^−4^). Additionally, 31 PRSs were significantly associated with the retreatment-free period with the PRS involving *ARMS2* and *CFH* again being most significantly associated (*p* = 1.12 × 10^−4^) as demonstrated by the Pearson correlation test (Figure 1).

Mean and median retreatment-free period was 7.4 ± 3.5 and 6 months, respectively. Eighty-six (33%) patients did not require additional injections. There was a significant negative correlation between the retreatment-free period and the polygenic risk score involving *ARMS2* and *CFH* (*R* = −0.237, *P* = 1.12 × 10^−4^, Pearson correlation test).

Moreover, we employed a multivariate regression analysis regarding the retreatment-free period. Of the 63 PRSs, 36 PRSs were significantly associated with retreatment-free period and the PRSs involving *ARMS2* and *CFH* were most significantly associated (*p* = 1.8 × 10^−5^, Table 5). After adjusting for age, gender, baseline BCVA, and subtypes, *ARMS2* solely was significantly associated with the retreatment and retreatment-free period (*p* = 1.6 × 10^−4^ and 8.4 × 10^−4^, multivariate regression analysis) and *CFH* solely was also associated with the retreatment and retreatment-free period (*p* = 0.03 and 0.01, multivariate regression analysis). Therefore, it might be reasonable that the PRSs involving *ARMS2* and *CFH* were most significantly associated.

During the twelve-month follow-up period, the mean number of additional required injections was 2.1 ± 2.1. The baseline factors associated with the number of additional required injections were shown in Table 5. Of the 63 possible combinations, 40 PRSs were significantly associated with the number of additional required injections during the study period, with the PRS involving *ARMS2* and *CFH* again being most significantly associated (*p* = 2.42 × 10^−6^).

## 3. Discussion

In daily clinical practice, persistent subretinal fluid on spectral domain optical coherence tomography (SD-OCT) despite monthly IAI is common, while only three loading phase injections achieve sustained macular dryness. Several clinical biomarker candidates have been investigated for an association with treatment response to neovascular AMD and PCV [12,13,14,15]. Given the reported association between genetic variants and various clinical phenotypes in exudative AMD [16,17,18,19,20], it is reasonable to hypothesize that genetic factors may be associated with response to intravitreal injection of VEGF inhibitors, including aflibercept. The present study quantified 63 PRSs to determine the potential association with one-year outcomes following as-needed aflibercept therapy exudative AMD. Results indicate that PRSs involving a combination of known high-risk variants were indeed associated with several treatment outcomes, including the need for additional injection, the number of additional required injections and BCVA at twelve months.

Previous pharmacogenetic studies, including ours, investigated whether a single variant was associated with treatment response to exudative AMD and concluded that risk variants of *ARMS2* were significantly associated with the need for additional injections and the number of additional required injections [21,22,23,24,25]. In the present study, variants of *ARMS2* A69S were the highest-risk variant among six variants examined. Even on its own, this variant was associated with the need for retreatment and number of additional required injections. However, relative to the ARMS2 variant in isolation, the PRSs with a combination of *ARMS2, CFH* and other genetic variants were more strongly associated with treatment outcomes, including the need for retreatment, the number of additional required injections, and BCVA at twelve months. These results indicate that PRSs involving a combination with high-risk genetic variants are better able to predict the treatment response than a single variant. Previous studies demonstrated that age is associated with retreatment/recurrence after initial treatment in exudative AMD [21,26]. As shown in Table 3, the hazard ratio (2.09) of the PRS incorporating *ARMS2* and *CFH* in predicting the requirement for retreatment is comparable to that provided by ten/eleven years of age advancement (1.07^11^ = 2.10). Age should thus be considered a major contributor to retreatment, even independent of PRSs.

Recently, PRSs have been utilized in the context of glaucoma. In primary open-angle glaucoma, a higher PRS is associated with several endophenotypes, including earlier disease onset, intraocular pressure, and optic disc vertical cup-to-disc ratio [27,28,29,30]. However, few reports demonstrated the association of a PRS with endophenotypes in exudative AMD [11]. The present study demonstrated that PRSs can be informative in predicting the treatment response in the context of exudative AMD, though further studies are required to elucidate the exact relationship between such PRSs, and clinical features and treatment response.

Potential imitations of the study include retrospective study design, relatively small sample size and genotyping only for a subset of variants susceptible to AMD. The second limitation is that we used the JSNP data as the data of controls. These data might not represent Japanese healthy controls. A large-scale prospective and genome-wide association study would be needed to confirm the current findings.

In summary, this is the first study to report the association between PRSs and treatment response in exudative AMD. Particularly, PRSs involving a combination with high-risk genetic variants were associated with treatment response, including the need for retreatment, the number of retreatments and BCVA at twelve months.

## 4. Materials and Methods

This retrospective study included consecutive patients with exudative AMD, including typical neovascular AMD and polypoidal choroidal vasculopathy (PCV) diagnoses, that were referred to the Macula Clinic, Ophthalmology, the University of Yamanashi between January 2013 and July 2019. This study was approved by the Ethics Committee and Institutional Review Board of the University of Yamanashi. It was conducted following the tenets of the Declaration of Helsinki 1975, as revised in 2000. Written informed consent was obtained from all patients to participate in this study.

### 4.1. Subjects

Medical records of consecutive patients meeting inclusion criteria and exclusion criteria were retrospectively reviewed. Inclusion criteria were as follows: (1) age greater than 50 years; (2) a diagnosis of typical neovascular AMD or PCV; (3) as-needed intravitreal aflibercept (0.2 mg/0.05 mL) injection (IAI) following 3 monthly IAIs; (4) completion of a twelve-month follow-up. Exclusion criteria are as follows: (1) other exudative maculopathies such as retinal angiomatous proliferation, angioid streaks and high myopia; (2) use of alternate treatment regimens such as fix-interval regimen and “treat and extend” regimen. Patients receiving other forms of treatment such as cataract surgery and photodynamic therapy were also excluded.

### 4.2. Follow-Up

Prior to the treatment, all patients received comprehensive ophthalmic examination such as decimal visual acuity test, intraocular pressure (IOP) measurement, slit-lamp with biomicroscope with or without 78 D lens, spectral-domain optical coherence tomography (SD-OCT) using Spectralis ver5.4 HRA + OCT (Heidelberg Engineering, Dossenheim, Germany) and fluorescein and indocyanine angiography (FA/ICGA). Both horizontal and vertical scans through the fovea were captured using SD-OCT at every visit. PCV was diagnosed when ICGA showed “hot spots”, which were hyperfluorescent, corresponding to retinal pigment epithelial protrusions, and neovascular AMD was diagnosed when leakage from the neovascular lesion was seen on FA without polypoidal lesion on ICGA and OCT showed type1 or type 2 neovascularization as previously described [31].

All patients received 3 monthly IAI and thereafter the monthly follow-up was performed. In each follow-up visit, BCVA and IOP measurements, biomicroscopic fundus examination and SD-OCT scans were performed. Additional IAI was required when SD-OCT demonstrated subretinal/intraretinal fluid or fundus examination demonstrated new subretinal or sub-RPE hemorrhage.

### 4.3. Genotyping and Calculation of PRSs

Peripheral venous blood was collected for baseline FA/ICGA. Genomic DNA was purified using a Pure Gene DNA Isolation Kit (Gentra Systems, Minneapolis, MN, USA). Patients were genotyped for six variants from six genes including *ARMS2* A69S (rs10490924), *CFH* I62V (rs800292), *C2-CFB-SKIV2L* (rs429608), *C3* (rs2241394), *CETP* (rs3764261), and *ADAMTS9* (rs679573) using TaqMan genotyping assays with 7300/7500 real-time PCR systems (Applied Biosystems, Foster City, CA, USA). Genotypic data of 172 healthy Japanese individuals were available from JSNP to serve as control data (demographic data were not available). A PRS was constructed by summing up the number of risk alleles of each single nucleotide polymorphism, weighted by their reported effect sizes (log odds ratio). Based on various variants combination, a total of 63 PRSs were calculated (_6_C_1_ + _6_C_2_ + _6_C_3_ + _6_C_4_ + _6_C_5_ + _6_C_6_).

### 4.4. Statistical Analysis

Statistical analyses were performed using DR. SPSS (IBM, Tokyo, Japan). Decimal BCVA was converted to a logMAR unit for statistical analysis. Differences of categorical and continuous variables between two groups were tested using the chi-square test and the Mann–Whitney U test, respectively. Multiple regression analysis was employed to reveal whether a PRS is associated with BCVA at 12 months. Multivariate logistic regression analysis was performed and correlated with the risk of retreatment with demographic factors and PRSs. A *p*-value of less than 0.05 was defined as a statistical significance.

## Figures and Tables

**Table 1:** Demographic and genetic characteristics of patients with exudative age-related macular degeneration (AMD).

Baseline Characteristics	Total	AMD (*n* = 129)	PCV(*n* = 132)	*p*-value
Age (years)	74.8 ± 8.3	77.1 ± 8.1	72.5 ± 7.8	3.84 × 10^−6^
Male gender	188 (72%)	86 (66.7%)	102 (77.3%)	0.056
Baseline logMAR BCVA	0.42 ± 0.37	0.50 ± 0.38	0.35 ± 0.34	1.2610^−4^
*ARMS2* A69S(rs10490924) T allele frequency	63.2%	68.2%	58.3%	0.019
*CFH* I62V(rs800292) G allele frequency	75.3%	77.9%	72.7%	0.17
*C2-CFB-SKIV2L*(rs429608) G allele frequency	91.8%	94.6%	89.0%	0.02
*C3* (rs2241394) G allele frequency	94.3%	94.6%	89.4%	0.03
*CETP* (rs3764261) A allele frequency	19.7%	19.0%	20.5%	0.67
*ADAMTS9* (rs6795735) T allele frequency	86.8%	86.8%	86.7%	0.98

AMD: age-related macular degeneration, PCVpolypoidal choroidal vasculopathy.

**Table 2:** Odds ratio in 6 genetic variants.

Genetic Variants	Exudative AMD Risk Allele Frequency	Controls Risk Allele Frequency	Odds Ratio	95% CI
*ARMS2* A69S (rs10490924) T allele (risk allele) (T:G)	0.632 (330:192)	0.384 (132:212)	2.76	2.06–3.66
*CFH* I62V (rs800292) G allele (risk allele) (G:A)	0.753 (393:129)	0.599(206:138)	2.04	1.52–2.74
*C3* (rs2241394) G allele (risk allele) (G:C)	0.961 (492:30)	0.901 (310:34)	1.80	1.08–3.00
*ADAMTS9* (rs6795735) T allele (risk allele) (T:C)	0.885 (453:69)	0.791 (272:72)	1.74	1.21–2.50
*SKIV2L-C2-CFB* (rs429608) G allele (risk allele) (G:A)	0.936 (479:43)	0.884 (304:40)	1.47	0.93–2.33
*CETP* (rs3764261) A allele (risk allele) (A:T)	0.197 (103:419)	0.198 (68:286)	0.97	0.69–1.36

CI: confidence interval.

**Table 3:** Baseline factors influencing logarithm minimum angle resolution (logMAR) BCVA at 12 months (multiple regression analysis).

Variable	β-Coefficient	*P* Value
Age	0.0034	0.096
Male gender	0.012	0.75
Baseline VA	0.614	3.0 × 10^−31^
PRS involving *ARMS2, CFH, C3,* and *ADAMTS9*	0.041	0.046

PRS: polygenic risk score.

**Table 4:** Comparison of baseline characteristics between patients requiring retreatment and patients without requiring retreatment.

Variables	Requiring Retreatment (*n* = 175)	Without Retreatment (*n* = 86)	*p*-Value	Hazard Ratio	95% CI
Age (year)	76.0 ± 7.9	72.3 ± 8.6	2.3 × 10^−3^	1.07	1.03–1.11
Male gender (%)	126 (72.0%)	62 (72.1%)	0.99	1.32	0.71–2.49
Baseline logMAR BCVA	0.41 ± 0.37	0.44 ± 0.37	0.52	0.59	0.28–1.27
PRS involving *ARMS2* and *CFH*	3.9 ± 0.75	3.5 ± 0.78	1.6 × 10^−4^	2.09	1.45–3.03

CI: confidence interval, PRS: polygenic risk score.

**Table 5:** Baseline factors associated with number of additional injections (multiple regression analysis).

Variable	β-Coefficient	*p* Value
Age	0.021	0.17
Male gender	0.27	0.33
Baseline VA	–0.86	0.011
PRS involving *ARMS2* and *CFH*	0.75	2.42 × 10^−6^

PRS: polygenic risk score.
